# Whole Exome Sequencing in Psoriasis Patients Contributes to Studies of Acitretin Treatment Difference

**DOI:** 10.3390/ijms18020295

**Published:** 2017-01-29

**Authors:** Xingchen Zhou, Yijing He, Yehong Kuang, Jie Li, Jianglin Zhang, Mingliang Chen, Wangqing Chen, Juan Su, Shuang Zhao, Panpan Liu, Menglin Chen, Minxue Shen, Xiaoping Chen, Wu Zhu, Xiang Chen

**Affiliations:** 1 Department of Dermatology, XiangYa Hospital, Central South University, Changsha 410008, China; zhouxingchen117@163.com (X.Z.); yijing.he@foxmail.com (Y.H.); yh_927@126.com (Y.K.); xylijie@medmail.com.cn (J.L.); leozjl1010@126.com (J.Z.); chenmingliang@medmail.com.cn (Mi.C.); lanchen2008@163.com (W.C.); sujuanderm@csu.edu.cn (J.S.); shuangxy@sina.com (S.Z.); liupanpan91@hotmail.com (P.L.); mollychen249821543@163.com (Me.C.); shenmx1988@gmail.com (M.S.); 2 Institute of Clinical Pharmacology, Xiangya Hospital, Central South University, Changsha 410008, China; chenxp74@hotmail.com; 3 Hunan Key Laboratory of Skin Cancer and Psoriasis, Changsha 410008, China

**Keywords:** whole exome sequencing, genetic variation, psoriasis, acitretin

## Abstract

Psoriasis vulgaris is an immune-mediated inflammatory skin disease. Although acitretin is a widely used synthetic retinoid for moderate to severe psoriasis, little is known about patients’ genetics in response to this drug. In this study, 179 patients were enrolled in either the discovery set (13 patients) or replication set (166 patients). The discovery set was sequenced by whole exome sequencing and sequential validation was conducted in the replication set by MassArray assays. Four SNPs (single nucleotide polymorphisms) (rs1105223T>C in *CRB2*, rs11086065A>G in *ANKLE1*, rs3821414T>C in *ARHGEF3*, rs1802073 T>G in *SFRP4*) were found to be significantly associated with acitretin response in either co-dominant or dominant models via multivariable logistic regression analysis, while *CRB2* rs1105223CC (OR = 4.10, 95% CI = 1.46–11.5, *p* = 0.007) and *ANKLE1* rs11086065AG/GG (OR = 2.76, 95% CI = 1.42–5.37, *p* = 0.003) were associated with no response to acitretin after 8-week treatment. Meanwhile, ARHGEF3 rs3821414CT/CC (OR = 0.25, 95% CI = 0.10–0.68, *p* = 0.006) and SFRP4 rs1802073GG/GT (OR = 2.40, 95% CI, 1.23–4.70, *p* = 0.011) were associated with a higher response rate. Four new genetic variations with potential influences on the response to acitretin were found in this study which may serve as genetic markers for acitretin in psoriasis patients.

## 1. Introduction

Psoriasis is a common immunologically mediated inflammatory skin disease, characterized by abnormal T-cell activation and inadequate keratinocyte differentiation [1,2,3]. It affects 2%–3% of the world population, and its morbidity is still increasing in recent years [4]. The pathogenesis of psoriasis is complex and involves genetic, environmental, immunological, and even, neurologic factors [5,6,7]. Although great efforts have been made in elucidating the pathogenesis of the disease, the full mechanism is not completely understood.

Acitretin is a synthetic retinoid belonging to the family of retinoid analogs (RA) drugs, and is widely used in moderate to severe psoriasis patients. Acitretin was speculated to regulate the differentiation, proliferation and apoptosis of human epidermal keratinocytes. It is thought to function through interfering with the expression of epidermal growth factor genes [8]. Also, it is reported that acitretin exerted an important influence on Th1 and Th17 cells during the treatment of psoriasis vulgaris; it reduced Th1 and Th17 cell infiltration and attenuated their cytokines in the skin [9]. There is also evidence that acitretin has immunomodulatory properties by inhibiting dermal microvascular endothelial cells and neutrophil migration [10]. The responsiveness of acitretin in psoriasis is notoriously variable. As reported, the response rate of acitretin, defined as 75%, improved the psoriasis area severity index (PASI) (PASI 75) after 12 weeks of treatment by 46%–52% [11]. There is little research focused on the metabolic process of acitretin in vivo, and the mechanism of pharmacology in psoriasis is still unclear.

Few pharmacogenomic studies have focused on this agent so far. Polymorphisms of the apolipoprotein E gene (*APOE*) and the vascular endothelial growth factor gene (*VEGF*) have been evaluated as predictors of response to psoriasis patients treated with acitretin. The results revealed that *ApoE* protein variants did not have any utility as pharmacogenetic markers for predicting patients’ response to acitretin [12], and the genetic variant (−460T>C) of VEGF was associated with the response to acitretin in psoriatic patients [13]. However, these studies only focused on isolated polymorphisms within single genes relevant to acitretin metabolism and considerable variation exists across all genes involved in acitretin metabolism. Utilizing whole exome sequencing, we have investigated predictors of outcome to acitretin therapy across each relevant gene in the largest patient cohort studied to date.

## 2. Results

### 2.1. Clinical Features of the Psoriatic Patients in Difference Phase

Among the discovery and verification phases, the baseline age, gender and BMI (Body Mass Index) of the patients had no significant difference between the effective and ineffective groups (Table 1). There were also no significant differences with respect to BMI (23.96 ± 3.95 vs. 22.99 ± 3.75, *p* = 0.340), age (48 ± 16 vs. 42 ± 13, *p* = 0.233) and gender (*p* = 0.216) between the discovery and verification phases.

### 2.2. Whole Exome Sequencing Analysis

**Figure 1:**
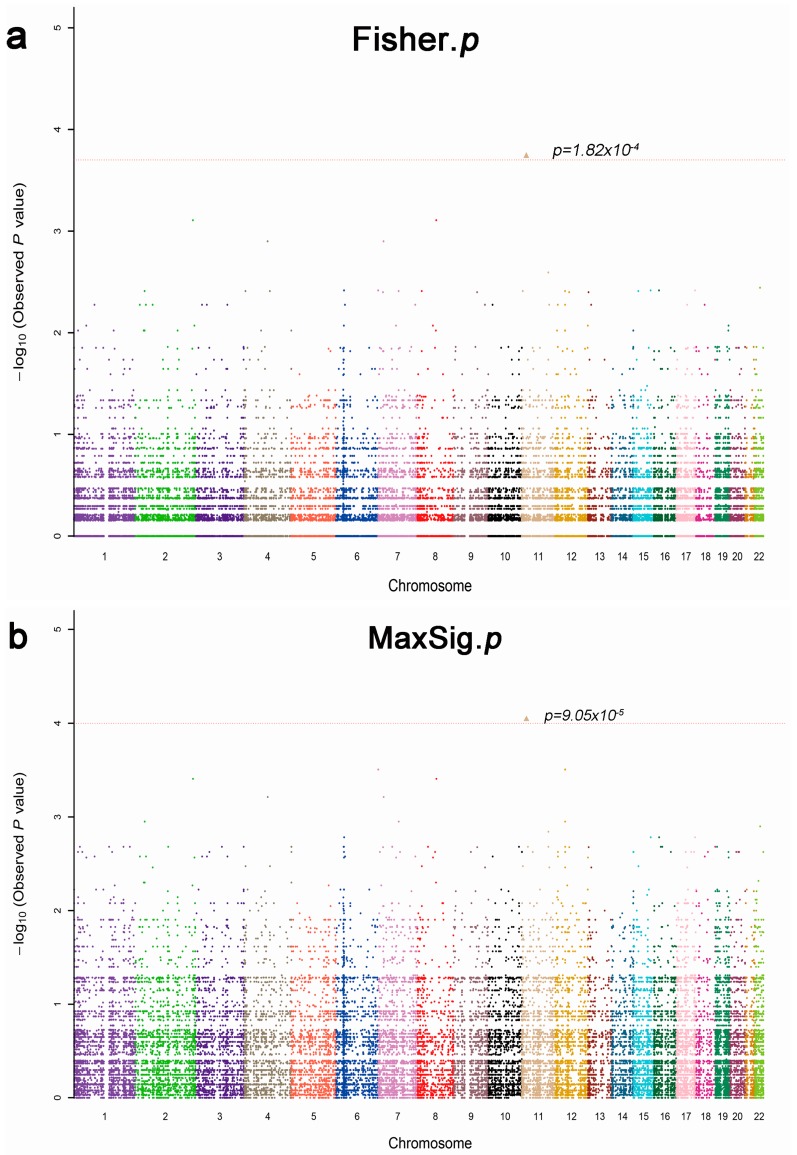
Manhattan plot of allele association tests of all SNPs (single nucleotide polymorphisms), that passed extreme phenotypes in 13 patients (five response and eight nonresponse). The different colors mean different chromosomes. (**a**) Fisher’s exact test, the most significant SNPs rs2241984, Fisher. *p* = 1.82 × 10^−4^; and (**b**) MaxSig. *p* is the most significant result in three methods of association analysis: Cochran–Armitage trend test, Fisher’s exact test, and different genetic models (dominant, recessive and general) test. The most significant SNPs rs2241984 MaxSig. *p* = 9.05 × 10^−5^.

**Figure 2:**
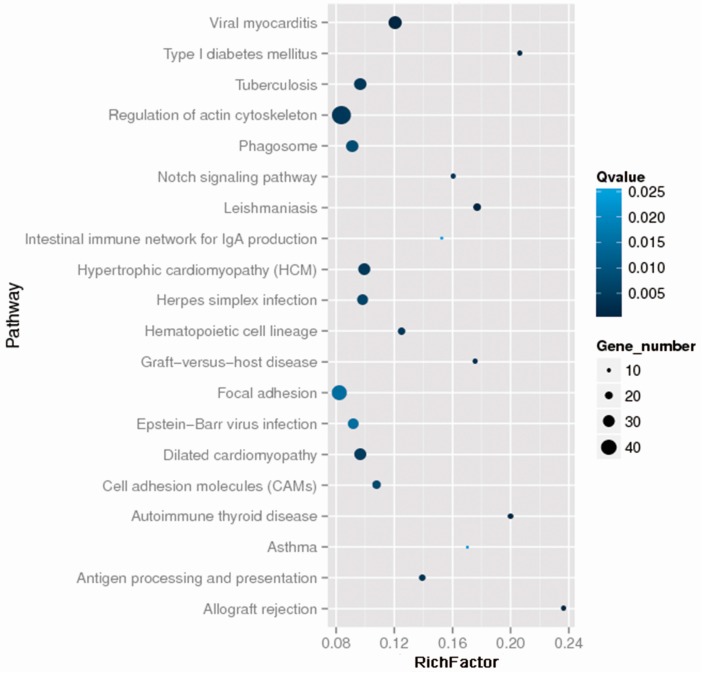
The top 20 statistics of Pathway Enrichment. The darker the color is, the more significant the Qvalue is; the Viral myocarditis pathway is the most significant among the top 20 statistics of Pathway Enrichment. The larger the circle area is, the higher the number of genes; the number of genes in the Regulation of the actin cytoskeleton pathway is the largest among the top 20 statistics of Pathway Enrichment.

To reconcile the clinical findings with molecular data in psoriatic patients, a total of 13 patients were selected for whole exome sequencing analysis, and we obtained 38,190 variants in this study. After identification of all the variant calls, Fisher’s exact test and the Cochran–Armitage trend as well as different genetic models (dominant, recessive and general) analyses were used to identify variants that were significantly associated with drug response (*p* < 0.05). In fact, 1790 variants were found to be associated with drug efficacy, the most significant SNPs rs2241984 (MaxSig. *p* = 9.04 × 10^−5^, Fisher. *p* = 1.82 × 10^−4^) as shown in Figure 1; and the top 20 statistics of Pathway Enrichment were shown in Figure 2. Moreover, according to the literature reports, mutation location and significance, 34 positive SNPs were selected and validated by MassArray in independent samples.

### 2.3. Univariate Analysis of Thirty-Four Positive SNPs

A total of 166 psoriatic patients were recruited in the verification phase. For quality control, only SNPs with a frequency above 5% and with a genotyping rate ≥95% were included in the final statistical analysis and 34 SNPs were all qualified. All SNPs were agreed with the Hardy–Weinberg equilibrium except for rs2303022, rs2376558, rs47 and rs76310711 variations. We then analyzed associations between the 34 selected SNPs and drug efficacy (Table 2). As shown in Table 2, we found that crumbs 2 (*CRB2*) rs1105223T>C, ankyrin repeat and LEM domain containing 1 (*ANKLE1*) rs11086065 A>G, Rho guanine nucleotide exchange factor 3 (*ARHGEF3*) rs3821414 T>C and secreted frizzled related protein 4 (*SFRP4*) rs1802073 G>T, were significantly associated with drug efficacy in either co-dominant or dominant models.

### 2.4. Multivariable Logistic Regression Analysis of CRB2 rs1105223T>C, ANKLE1 rs11086065 A>G, ARHGEF3 rs3821414 T>C and SFRP4 rs1802073 G>T

In order to further verify the effect of four SNPs, multivariable logistic regression analysis with adjustment for confounders including age, gender and body mass index (BMI) was used. For CRB2 rs1105223T>C variation, 63 patients carried the CRB2 rs1105223TT genotype, 64 patients carried the CRB2 rs1105223CT genotype, and 29 patients carried the CRB2 rs1105223CC genotype. CRB2 rs1105223CC was associated with the ineffective response (OR = 4.098, 95% CI = 1.461–11.493, *p* = 0.007) compared to the TT genotype, and CRB2 rs1105223TT/CT was also associated with the drug efficacy compared to the CC genotype (OR = 0.588, 95% CI, 0.363–0.955, *p* = 0.032).

For ANKLE1 rs11086065 A>G variation, 69 patients carried the ANKLE1 rs11086065AA genotype, 72 patients carried the ANKLE1 rs11086065 AG genotype, and 21 patients carried the ANKLE1 rs11086065GG genotype. ANKLE1 rs11086065AG/GG was associated with the ineffective response compared to the GG genotype (OR = 2.756, 95% CI, 1.415–5.368, *p* = 0.003) and the ANKLE1 rs11086065G allele was associated with the ineffective response (OR = 1.939, 95% CI, 1.171–3.210, *p* = 0.010).

For ARHGEF3 rs3821414 T>C variation, 69 patients carried the ARHGEF3 rs3821414TT genotype, 73 patients carried the ARHGEF3 rs3821414CT genotype and 24 patients carried the ARHGEF3 rs3821414CC genotype. ARHGEF3 rs3821414CT was associated with the effective response compared to the TT genotype (OR = 0.253, 95% CI, 0.095–0.675, *p* = 0.006) and the ARHGEF3 rs3821414C allele was associated with the effective response (OR = 0.487, 95% CI, 0.305–0.779, *p* = 0.003).

For SFRP4 rs1802073 G>T variation, 57 patients carried the SFRP4 rs1802073TT genotype, 88 patients carried the SFRP4 rs1802073GT genotype and 21 patients carried the SFRP4 rs1802073GG genotype. SFRP4 rs1802073GG/GT was associated with the effective response compared to the TT genotype (OR = 2.400, 95% CI, 1.226–4.696, *p* = 0.011) and the SFRP4 rs1802073T allele was associated with the effective response (OR = 0.612, 95% CI, 0.380–0.984, *p* = 0.043). The details were seen in Table 3.

## 3. Discussion

In this study, we performed whole exome sequencing for 13 psoriasis patients, who experienced either high or extremely low efficacy. Although *p* values obtained at the discovery stage were individually rather weak, they provide us some biological knowledge for reference. To gain further insight into the potential influence of these genetic markers on the outcome of acitretin treatment in patients with psoriasis, we picked 34 SNPs for validation in an independent set of 166 patients by Sequenom MassArray. The 34 SNPs (as shown in the Table 2) were primarily chosen from the pathway enrichment (Figure 2) and the results of the whole exome sequencing with *p* value <0.05. Several studies have previously proved the association between candidate genes involved in metabolic pathways of acitretin and the pathogenic mechanism of psoriasis, such as *CSMD1*, *CCHCR1*, *GLI1*, *SFRP4* etc. [14,15,16,17]. In this study, we identified four SNPs that might be associated with the response to acitretin. The four genetic variants—rs1802073 G>T in *SFRP4*, rs1105223T>C in *CRB2*, rs3821414T>C in *ARHGEF3*, rs11086065A>G in *ANKLE1—*could be validated as predictive markers for the response to acitretin in psoriasis.

Genetic-associated studies identified dozens of psoriasis associated genes and the signaling pathways [18,19] include Notch signaling and Wnt signaling [20,21]. Notch signaling is associated with normally differentiated human epidermis, confirming its involvement in keratinocyte differentiation [22]. Wnt signaling participates in cell proliferation, adhesion and differentiation, suggesting that this pathway might be involved in psoriasis pathogenesis [23,24]. Acitretin is a retinoic acid derivative, although the mechanisms of acitretin to treat psoriasis is unclear, it is possible that the inhibition of Wnt signaling and the activation of Notch signaling [25].

The genetic variant rs1802073 in *SFRP4* is a missense variant (a type of nonsynonymous substitution); rs1802073 at position g.7:37947164G>T results in a proline to threonine change at position 320 in the *SFRP4* protein. *SFRP4* is one of the secreted frizzled-related protein family members, thought to be a negative regulator of the Wnt signaling pathway [26,27]. Recently, it has been shown that the expression of *SFRP4* was diminished in the lesional skin of patients with psoriasis [27]. *SFRP4* directly inhibits the excessive keratinocyte proliferation evoked, and decreases the severity of the psoriasiform skin phenotype, including decreased acanthosis and reduced leukocyte infiltration [17]. Recently, Green et al indicated that *SFRP4* could be a direct target gene of RARs, and RAR agonist induced the significant upregulation of SFRP4 [28]. Furthermore, acitretin is a member of the RAR agonist family, so we speculated that acitretin may alter the transcriptional regulation of *SFRP4*, such as encoding soluble Wnt antagonists and also ligands and receptors of the Notch pathway [25,29].

The rs1105223 in *CRB2* is a missense variant; it is at position g.9:126128211T>C, resulting in a methionine to threonine shift at position 145 in the *CRB2* protein. *CRB2* is known to contain 15 extracellular EGF-like domains and three extracellular laminin G-like domains; it encodes the extracellular tenth EGF-like domain, and acts as an inhibitory binding protein to influence Notch signaling [30]. The rs11086065 in *ANKLE1* is a missense variant at position G.19: 17284194A>G, resulting in a glutamine to arginine shift at position 452 in the *ANKLE1* protein. Few references could be found about the function of *ANKLE1*, but because it contains a GIY-YIG-type (conserved N-terminal catalytic domains connected by linkers to C-terminal DNA-binding domains) nuclease domain, there is likely to be a potential role for *ANKLE1* in DNA damage response [31]. A recent study presented a potential linkage of SNPs in the human *ANKLE1* gene, showing an association between a function of *ANKLE1* in multiple autoimmune syndromes [32] and the increased risk of certain cancers [33]. The rs3821414 in *ARHGEF3* is a 3_prime_UTR_variant; this genetic variant does not result in amino acid change. One of the functions of *ARHGEF3* is that it modulates differentiation through the activation of RhoA [34]. Although the three SNPs were associated with the ineffective/effective response to acitretin, we cannot conclude whether these SNPs influence the response to acitretin.

In our study, the genetic variants (rs1802073, rs1105223, rs11086065) introduce amino acid changes and may affect protein function, so we used bioinformatics approaches to analyze the effect of these SNPs on protein structure and function, such as PolyPhen, SNPeffect, SIFT and GTEx Protal. No significant eQTLs were found for four SNPs (rs1802073, rs1105223, rs11086065, rs3821414) in tissue Whole Blood by using GTEx Protal. Furthermore, PolyPhen, SIFT and SNPeffect can predict the function of protein-coding: rs1802073T>G in *SFRP4* was judged to be possibly damaging and tolerated; rs1105223T>C in *CRB2* was judged to be benign and tolerated; rs11086065A>G in *ANKLE1* was judged to be benign and deleterious, respectively. Therefore, it is generally believed that these SNPs may influence the response to acitretin, and further study is needed on the mechanism.

## 4. Materials and Methods

### 4.1. Patients

This study was approved by the Ethic Committee of XiangYa Hospital, the registration number of Chinese Clinical Trial Registry online is ChiCTR-OCH-14004518, and the registration number of ClinicalTrials.gov Protocol Registration and Results System (PRS) is NCT02715960. From April 2014 to July 2016, a total of 179 psoriatic patients (13 patients in the discovery set and 166 for the replication set) with moderate to severe psoriasis were recruited in the Department of Dermatology, Xiang Ya Hospital, Central South University. In the discovery set, five patients were defined as the responders with 100% improvement of PASI from the baseline post and eight patients were non-responders with −20% to −200% improvement of PASI (mean more serious) from the baseline post at 12 weeks of treatment. In the replication set, 100 patients (60.2%) were responders who achieved PASI75 at week 12, and 66 patients (39.8%) were non-responders who failed PASI75. The demographic, photography data as well as clinical data of psoriatic patients at each visit were collected. All patients were treated by a combination of 30 mg/day acitretin (Huapont Pharm., Chongqing, China) and a topical cream, calcipotriol (Bright Future Pharmaceutical Laboratories Ltd., Hongkong) for 12 weeks before the first follow-up.

The psoriatic patients who participated in this study were from the Southern Han Chinese population with the inclusion criteria as follows: (i) patients fulfilled the diagnostic criteria for psoriasis vulgaris; (ii) patients were aged ≥18 years; (iii) no medicines were received in the last four weeks before administration of the study agent. The exclusion criteria were as follows: (i) had other dermatological problems or any other diseases, and required pharmacological treatment; (ii) pregnant women, breastfeeding mothers, or women who were trying to become pregnant within the next 3 years. The study was approved by the institutional review board of Xiang Ya Hospital, Changsha, China. The written informed consent was obtained from each patient before participating in this study. The severity of psoriasis and the drug efficacy were both assessed by PASI. Patients with a PASI score greater than 10 are defined as moderate to severe psoriasis [35]. In conformity with guidelines, PASI75 at week 12 after the initiation of treatment was adopted as the index of response to acitretin in this study, which corresponds to a 75% improvement from baseline. Patients who reached a reduction of less than 75% from the baseline PASI at week 12 were considered as nonresponders.

### 4.2. DNA Extraction

Genomic DNA was extracted from the venous blood samples (5 mL) using a FlexiGene DNA Kit for mammalian blood (Qiagen, Hilden, Germany) according to manufacturer’s instructions. Purity/concentration was determined using a Bio-spec Nano Spectrophotometer (Shimadzu Corporation, Japan). All the blood samples were stored at −80 °C until used.

### 4.3. Whole Exome Sequencing

Each captured library was then loaded on the Illumina Hiseq2000 platform (Illumina, San Diego, CA, USA). Whole exome sequencing was performed using Illumina base-calling Software 1.7 for base calling with default parameters. Briefly, DNA was cut into as well as purified 200–300-bp fragments. DNA was then amplified by PCR; clusters of PCR colonies were then sequenced on the Illumina Hiseq2000 platform (Illumina, USA); the sequence of each individual was generated from paired-end 90-bp reads.

The raw sequence data were aligned to the GRCh37 human reference genome using Burrows–Wheeler Aligner (BWA v0.7.7-r411) [36]. PCR duplicates were marked using the Mark Duplicates program in Picard-tools-1.115 tool set. GATK v3.2-2 and Samtools were used for the identification of INDEL, base quality score recalibration (BQSR) and the SNVs (single nucleotide variants) respectively. All variants were annotated using the Annovar program. The Mapping Quality Rank Sum Test (MQRankSum, *u*-based *z*-approximation from the Mann–Whitney Rank Sum Test for mapping qualities, only for heterozygous calls) and Read Pos Rank Sum (*u*-based *z*-approximation score from the Mann–Whitney Rank Sum Test for the distance from the end of the read for reads with the alternate allele, only for heterozygous calls) were conducted. The basic association tests implemented are the Cochran–Armitage trend test, Fisher's exact test, and different genetic models (dominant, recessive and general). Fisher's exact test was used to perform a standard case/control association analysis to generate significance. Those significance mutations are selected for annotation when the *p* value <0.05.

### 4.4. Sequenom MassArray Analysis

Thirty-four SNPs (single nucleotide polymorphisms) were analyzed using Sequenom MassArray (Sequenom, San Diego, CA, USA) according to the standard protocol recommended by the manufacturer [37].

### 4.5. Data Statistics and Analysis

The entire analysis was performed in the SPSS 23.0 statistical package (IBM SPSS, Chicago, IL, USA). The allele frequencies in different subgroups were tested by the chi-square analysis method. The chi-square test was used to determine whether genotype distribution of the SNPs agreed with Hardy–Weinberg equilibrium and to compare the distribution of categorical variables between drug response groups. The Fisher’s exact test was used when data were spare. Comparisons of continuous variables between or among genotype groups were performed using nonparametric tests (Mann–Whitney *U* test). Adjusted odds ratio (ORs) and 95% confidence intervals (CIs) were used to describe drug outcomes. OR and 95% CI were calculated by limited backward-LR (likelihood ratio) logistic regression analysis with adjustment by clinical variables. A two-tailed *p* value less than 0.05 was regarded to be significant. The post hoc power of the sample size in χ-square analysis was operated with G. power (versions 3.1.9.2): the effect size was 1.024 and 0.417; α error was 0.05, and df was 5; and the sample size was 45 and 105 in the discovery and verification phases respectively, thus the power presented from 0.999 to 1.000.

## 5. Conclusions

In conclusion, four SNPs (rs1802073T>G in SFRP4, rs1105223T>C in CRB2, rs11086065A>G in ANKLE1, rs3821414T>C in ARHGEF3) were found to be associated with acitretin response via whole exome sequence and sequential validation, and there is accumulating evidence regarding the functional effects of these SNPs, especially rs1802073T>G in SFRP4. In the future, with additional work and validation, these variants will prove to be useful as markers for targeting therapies; they will be applicable more precisely and safely to individual patients, to optimize the treatment of psoriasis and minimize unnecessary expenditure.

## Figures and Tables

**Table 1:** The demographic data of the patients in two phases.

Characteristics	Discovery Phase (*n* = 13)		*p* Value	Verification Phase (*n* = 166)		*p* Value
	PASI < 75	PASI ≥ 75		PASI < 75	PASI ≥ 75	
Age (mean ± SD)	45 ± 12	52 ± 22	0.284	41 ± 13	43 ± 13	0.562
Gender: Male, *n* (%)	3 (37.5)	4 (80)	0.266 ^1^	66 (66)	52 (78.8)	0.075
Female, *n* (%)	5 (62.5)	1 (20)		34 (34)	14 (21.2)	
BMI (mean ± SD)	24.31 ± 4.44	23.25 ± 3.21	0.683	22.93 ± 3.90	23.08 ± 3.53	0.841

^1^ Fisher’s exact test; PASI: Psoriasis Area and Severity Index; BMI: Body Mass Index; SD: standard deviation.

**Table 2:** Univariate analysis of thirty-four positive SNPs.

Number	SNP ID	Gene	Chr	MAF	Location	Genotype	*n*	*p* for H–W	*p* Value (Drug Response)		
									Codominant	Dominant	Recessive
1	rs10097933	*CSMD1*	8	0.14	Intron_variant	TT/CT/CC	116/46/3	0.52	0.895	0.650	0.828
2	rs10775247	*TICRR*	15	0.34	Missense_variant	CC/CT/TT	73/80/12	0.11	0.162	0.087	0.701
3	rs1105223	*CRB2*	9	0.47	Missense_variant	TT/CT/CC	63/64/29	0.08	***0.015***	0.497	***0.020***
4	rs11086065	*ANKLE1*	19	0.29	Missense_variant	AA/AG/GG	69/72/21	0.74	***0.017***	***0.004***	0.272
5	rs1142825	*CALML3*	10	0.49	Synonymous_variant	AA/AG/GG	61/83/22	0.45	0.919	0.680	0.906
6	rs11674608	*CHRNG*	2	0.42	Upstream_gene_variant	GG/CG/CC	57/79/29	0.86	0.698	0.789	0.504
7	rs13026692	*ALPP*	2	0.46	Missense_variant	TT/AT/AA	45/85/36	0.73	0.849	0.693	0.792
8	rs1802073	*SFRP4*	7	0.46	Missense_variant	TT/GT/GG	57/88/21	0.15	***0.021***	***0.005***	0.520
9	rs2075333	*TSPAN11*	12	0.22	Missense_variant	CC/CT/TT	75/78/13	0.24	0.878	0.794	0.624
10	rs2076015	*TMX4*	20	0.38	Missense_variant	TT/CT/CC	62/83/21	0.40	0.607	0.588	0.520
11	rs2235638	*IFT140*	16	0.17	Missense_variant	GG/AG/AA	97/59/10	0.80	0.514	0.269	0.515
12	rs2241984	*PTPN5*	11	0.22	Intron_variant	GG/AG/AA	114/46/6	0.62	0.938	0.818	0.743
13	rs2303022	*ANXA6*	5	0.48	Intron_variant	GG/CG/CC	39/97/30	**0.03**	0.531	0.573	0.427
14	rs2303694	*ELL*	19	0.10	Missense_variant	CC/CT/TT	132/33/1	0.49	0.682	0.850	0.415
15	rs2376558	*TPCN2*	11	0.48	Missense_variant	CC/CT/TT	45/94/26	**0.05**	0.367	0.284	0.541
16	rs2547065	*MUC16*	19	0.21	Missense_variant	GG/CG/CC	107/55/4	0.32	0.790	0.875	0.541
17	rs2933352	*MUC19*	12	0.26	Missense_variant	TT/CT/CC	91/61/14	0.41	0.660	0.487	0.747
18	rs2933353	*MUC19*	12	0.31	Missense_variant	CC/AC/AA	74/73/19	0.88	0.875	0.615	0.782
19	rs322118	*COL6A5*	3	0.16	Splice_region_variant	AA/AG/GG	103/54/7	0.98	0.191	0.351	0.079
20	rs335824	*NCBP2*	3	0.24	Upstream_gene_variant	TT/CT/CC	91/67/8	0.33	0.259	0.562	0.178
21	rs3733160	*TBCCD1*	3	0.11	Upstream_gene_variant	GG/AG/AA	122/42/1	0.19	0.715	0.942	0.413
22	rs3741595	*ORAI1*	12	0.18	Synonymous_variant	CC/CT/TT	80/73/12	0.40	0.428	0.428	0.435
23	rs3748664	*HHIPL2*	1	0.50	Synonymous_variant	CC/CG/GG	42/89/34	0.30	0.397	0.868	0.181
24	rs3817475	*GLI1*	12	0.29	Intron_variant	AA/AG/GG	96/57/13	0.28	0.797	0.789	0.642
25	rs3821414	*ARHGEF3*	3	0.36	3_Prime_UTR_variant	TT/CT/CC	69/73/24	0.51	***0.006***	***0.002***	***0.044***
26	rs386624809	*SLC36A3*	5	0.42	Missense_variant	CC/CT/TT	44/92/30	0.13	0.927	0.856	0.702
27	rs47	*THSD7A*	7	0.25	Missense_variant	CC/CT/TT	82/77/6	**0.02**	0.373	0.923	0.373
28	rs56310840	*GBA*	1	0.25	Downstream_gene_variant	AA/AG/GG	82/64/19	0.24	0.340	0.239	0.215
29	rs7133914	*LRRK2*	12	0.10	Missense_variant	GG/AG/AA	120/24/3	0.19	0.948	0.744	0.921
30	rs7146310	*IPO4*	14	0.40	Missense_variant	GG/AG/AA	60/76/30	0.49	0.592	0.479	0.659
31	rs72927138	*LIN54*	4	0.49	5_Prime_UTR_variant	GG/AG/AA	45/74/42	0.31	0.360	0.449	0.398
32	rs74976577	*ISYNA1*	19	0.17	Downstream_gene_variant	GG/GT/TT	101/59/6	0.46	0.486	0.356	0.602
33	rs76310711	*TNXB*	6	-	Missense_variant	CC/CG/GG	57/92/14	**0.01**	0.543	0.360	0.418
34	rs916235	*C1QTNF6*	22	0.45	3_Prime_UTR_variant	TT/CT/CC	50/84/32	0.76	0.769	0.516	0.608

SNP: single nucleotide polymorphism; Chr: chromosome; MAF: minor allele frequency; H–W: Hardy–Weinberg equilibrium; missense variant: a type of non-synonymous substitution. Bold and italics in *p* Value mean the result is significant; and bold in *p* for H–W mean the result is not consistent with Hardy–Weinberg equilibrium.

**Table 3:** Multivariate logistic regression analysis of four positive SNPs.

Gene	SNPs	Genotypes/Alleles	PASI < 75	PASI ≥ 75	Adjusted OR ^1^ [95% CI]	*p* Value
			*n* = 100	*n* = 66		
*CRB2*	rs1105223	TT, *n* (%)	40 (42.6)	23 (37.1)	1.00	
		CT, *n* (%)	31 (33)	33 (53.2)	2.012 [0.710–5.706]	0.188
		CC, *n* (%)	23 (24.4)	6 (9.7)	4.098 [1.461–11.493]	***0.007***
		CT/CC, *n* (%)	54 (57.4)	39 (62.9)	1.363 [0.679–2.735]	0.383
		TT/CT, *n* (%)	71 (75.5)	56 (90.3)	0.588 [0.363–0.955]	***0.032***
		T, *n* (%)	111 (59)	79 (63.7)	1.00	
		C, *n* (%)	77 (41)	45 (36.3)	1.118 [0.687–1.820]	0.652
*ANKLE1*	rs11086065	AA, *n* (%)	33 (33.7)	36 (56.3)	1.00	
		AG, *n* (%)	50 (51)	22 (34.3)	2.552 [0.876–7.439]	0.086
		GG, *n* (%)	15 (15.3)	6 (9.4)	0.905 [0.303–2.700]	0.858
		AG/GG, *n* (%)	65 (66.3)	28 (43.8)	2.756 [1.415–5.368]	***0.003***
		AA/AG, *n* (%)	83 (84.7)	58 (90.6)	0.815 [0.485–1.369]	0.439
		A, *n* (%)	116 (59.2)	94 (73.4)	1.00	
		G, *n* (%)	80 (40.8)	34 (26.6)	1.939 [1.171–3.210]	***0.010***
*ARHGEF3*	rs3821414	TT, *n* (%)	51 (51)	18 (27.3)	1.00	
		CT, *n* (%)	39 (39)	34 (51.5)	0.253 [0.095–0.675]	***0.006***
		CC, *n* (%)	10 (10)	14 (21.2)	0.568 [0.222–1.451]	0.237
		CT/CC, *n* (%)	49 (49)	48 (72.7)	0.386 [0.194–0.765]	***0.006***
		TT/CT, *n* (%)	90 (90)	52 (78.8)	1.593 [1.024–2.479]	***0.039***
		T, *n* (%)	141 (70.5)	70 (53)	1.00	
		C, *n* (%)	59 (29.5)	62 (47)	0.487 [0.305–0.779]	***0.003***
*SFRP4*	rs1802073	GG, *n* (%)	14 (14)	7 (10.6)	1.00	
		GT, *n* (%)	60 (60)	28 (42.4)	0.483 [0.167–1.394]	0.178
		TT, *n* (%)	26 (26)	31 (47)	0.402 [0.199–0.812]	***0.011***
		GT/TT, *n* (%)	86 (86)	59 (89.4)	0.797 [0.296–2.149]	0.797
		GG/GT, *n* (%)	74 (74)	35 (53)	2.400 [1.226–4.696]	***0.011***
		G, *n* (%)	88 (44)	42 (31.8)	1.00	
		T, *n* (%)	112 (56)	90 (68.2)	0.612 [0.380–0.984]	***0.043***

^1^ adjusted for age, gender and BMI. Bold and italics in *p* value mean the significant result.
