# Correction: Xingchen Zhou, et al. Whole Exome Sequencing in Psoriasis Patients Contributes to Studies of Acitretin Treatment Difference. *Int. J. Mol. Sci.* 2017, *18*, 295

**DOI:** 10.3390/ijms18091899

**Published:** 2017-09-04

**Authors:** Xingchen Zhou, Yijing He, Yehong Kuang, Jie Li, Jianglin Zhang, Mingliang Chen, Wangqing Chen, Juan Su, Shuang Zhao, Panpan Liu, Menglin Chen, Minxue Shen, Xiaoping Chen, Wu Zhu, Xiang Chen

**Affiliations:** 1 Department of Clinical Pharmacology, Xiangya Hospital, Central South University, Changsha 410008, China; zhouxingchen117@163.com (X.Z.); chenxp74@hotmail.com (X.C.); 2 Department of Dermatology, Xiangya Hospital, Central South University, Changsha 410008, China; yijing.he@foxmail.com (Y.H.); yh_927@126.com (Y.K.); xylijie@medmail.com.cn (J.L.); leozjl1010@126.com (J.Z.); chenmingliang@medmail.com.cn (M.C.); lanchen2008@163.com (W.C.); sujuanderm@csu.edu.cn (J.S.); shuangxy@sina.com (S.Z.); liupanpan91@hotmail.com (P.L.); mollychen249821543@163.com (M.C.); shenmx1988@gmail.com (M.S.); 3 Hunan Key Laboratory of Skin Cancer and Psoriasis, Changsha 410008, China

We would like to submit the following correction to the published paper [1], the reason for this action is that the data in Table 3 were reanalyzed by one more accurate statistic method: On page 12, the sentence of paragraph three “OR and 95% CI were calculated by limited backward-LR (likelihood ratio) logistic regression analysis with adjustment by clinical variables” should be corrected into “OR and 95% CI were calculated by limited enter logistic regression analysis with adjustment by clinical variables”. The corrections resulted some changes in Table 3 and related data in text, while the significance and the conclusions remained unchanged. Furthermore, in order to make the reader easy to understand the data in Table 3, we revised the table title and added notes for Table 3 (page 7).

Table 3 should be replaced:

**Table 3:** Multivariate logistic regression analysis of four positive SNPs.

Gene	SNPs	Genotypes/Alleles	PASI < 75	PASI ≥ 75	Adjusted OR ^1^ [95% CI]	*p* Value
			*n* = 100	*n* = 66		
*CRB2*	rs1105223	TT, *n* (%)	40 (42.6)	23 (37.1)	1.00	
		CT, *n* (%)	31 (33)	33 (53.2)	2.012 [0.710–5.706]	0.188
		CC, *n* (%)	23 (24.4)	6 (9.7)	4.098 [1.461–11.493]	***0.007***
		CT/CC, *n* (%)	54 (57.4)	39 (62.9)	1.363 [0.679–2.735]	0.383
		TT/CT, *n* (%)	71 (75.5)	56 (90.3)	0.588 [0.363–0.955]	***0.032***
		T, *n* (%)	111 (59)	79 (63.7)	1.00	
		C, *n* (%)	77 (41)	45 (36.3)	1.118 [0.687–1.820]	0.652
*ANKLE1*	rs11086065	AA, *n* (%)	33 (33.7)	36 (56.3)	1.00	
		AG, *n* (%)	50 (51)	22 (34.3)	2.552 [0.876–7.439]	0.086
		GG, *n* (%)	15 (15.3)	6 (9.4)	0.905 [0.303–2.700]	0.858
		AG/GG, *n* (%)	65 (66.3)	28 (43.8)	2.756 [1.415–5.368]	***0.003***
		AA/AG, *n* (%)	83 (84.7)	58 (90.6)	0.815 [0.485–1.369]	0.439
		A, *n* (%)	116 (59.2)	94 (73.4)	1.00	
		G, *n* (%)	80 (40.8)	34 (26.6)	1.939 [1.171–3.210]	***0.010***
*ARHGEF3*	rs3821414	TT, *n* (%)	51 (51)	18 (27.3)	1.00	
		CT, *n* (%)	39 (39)	34 (51.5)	0.253 [0.095–0.675]	***0.006***
		CC, *n* (%)	10 (10)	14 (21.2)	0.568 [0.222–1.451]	0.237
		CT/CC, *n* (%)	49 (49)	48 (72.7)	0.386 [0.194–0.765]	***0.006***
		TT/CT, *n* (%)	90 (90)	52 (78.8)	1.593 [1.024–2.479]	***0.039***
		T, *n* (%)	141 (70.5)	70 (53)	1.00	
		C, *n* (%)	59 (29.5)	62 (47)	0.487 [0.305–0.779]	***0.003***
*SFRP4*	rs1802073	GG, *n* (%)	14 (14)	7 (10.6)	1.00	
		GT, *n* (%)	60 (60)	28 (42.4)	0.483 [0.167–1.394]	0.178
		TT, *n* (%)	26 (26)	31 (47)	0.402 [0.199–0.812]	***0.011***
		GT/TT, *n* (%)	86 (86)	59 (89.4)	0.797 [0.296–2.149]	0.797
		GG/GT, *n* (%)	74 (74)	35 (53)	2.400 [1.226–4.696]	***0.011***
		G, *n* (%)	88 (44)	42 (31.8)	1.00	
		T, *n* (%)	112 (56)	90 (68.2)	0.612 [0.380–0.984]	***0.043***

^1^ adjusted for age, gender and BMI. Bold and italics in *p* value mean the significant result.

with

**Table 3:** Association of four positive SNPs with response to acitretin via logistic regression analysis.

Gene	SNPs	Genotypes/Alleles	PASI < 75	PASI ≥ 75	Adjusted OR ^1^ [95% CI]	*p* Value
			*n* = 100	*n* = 66		
*CRB2*	rs1105223 ^a^	TT, *n* (%)	40 (42.6)	23 (37.1)	1.00	
		CT, *n* (%)	31 (33)	33 (53.2)	0.498 [0.234–1.062]	0.071
		CC, *n* (%)	23 (24.4)	6 (9.7)	1.852 [0.640–5.359]	0.256
		CT/CC, *n* (%)	54 (57.4)	39 (62.9)	0.734 [0.366–1.472]	0.383
		TT/CT, *n* (%)	71 (75.5)	56 (90.3)	0.371 [0.139–1.085] ^2^	***0.048***
		T, *n* (%)	111 (59)	79 (63.7)	1.00	
		C, *n* (%)	77 (41)	45 (36.3)	1.118 [0.687–1.820]	0.652
*ANKLE1*	rs11086065 ^b^	AA, *n* (%)	33 (33.7)	36 (56.3)	1.00	
		AG, *n* (%)	50 (51)	22 (34.3)	2.922 [1.413–6.041]	***0.004***
		GG, *n* (%)	15 (15.3)	6 (9.4)	2.553 [0.851–7.652]	0.094
		AG/GG, *n* (%)	65 (66.3)	28 (43.8)	2.835 [1.436–5.600]	***0.003***
		AA/AG, *n* (%)	83 (84.7)	58 (90.6)	0.664 [0.235–1.875] ^3^	0.439
		A, *n* (%)	116 (59.2)	94 (73.4)	1.00	
		G, *n* (%)	80 (40.8)	34 (26.6)	1.966 [1.181–3.271]	***0.009***
*ARHGEF3*	rs3821414	TT, *n* (%)	51 (51)	18 (27.3)	1.00	
		CT, *n* (%)	39 (39)	34 (51.5)	0.464 [0.222–0.968]	***0.041***
		CC, *n* (%)	10 (10)	14 (21.2)	0.256 [0.098–0.713]	***0.009***
		CT/CC, *n* (%)	49 (49)	48 (72.7)	0.402 [0.201–0.805]	***0.01***
		TT/CT, *n* (%)	90 (90)	52 (78.8)	2.465 [1.011–6.013] ^4^	***0.047***
		T, *n* (%)	141 (70.5)	70 (53)	1.00	
		C, *n* (%)	59 (29.5)	62 (47)	0.497 [0.310–0.797]	***0.004***
*SFRP4*	rs1802073	GG, *n* (%)	14 (14)	7 (10.6)	1.00	
		GT, *n* (%)	60 (60)	28 (42.4)	1.196 [0.421–3.396]	0.737
		TT, *n* (%)	26 (26)	31 (47)	0.449 [0.153–1.321]	0.146
		GT/TT, *n* (%)	86 (86)	59 (89.4)	0.797 [0.296–2.149]	0.655
		GG/GT, *n* (%)	74 (74)	35 (53)	2.570 [1.294–5.107] ^5^	***0.007***
		G, *n* (%)	88 (44)	42 (31.8)	1.00	
		T, *n* (%)	112 (56)	90 (68.2)	0.603 [0.374–0.971]	***0.037***

^1^ adjusted for age, gender and BMI, OR < 1 means response to acitretin, OR > 1 means non-response to acitretin; ^2^ compared to the CC genotype; ^3^ compared to the GG genotype; ^4^ compared to the CC genotype; ^5^ compared to the TT genotype; ^a^ total of 156 samples were detected; ^b^ total of 162 samples were detected; Bold and italics in *p* value mean the significant result.

In addition, there were some writing errors: “rs1802073T>G” should be corrected into “rs1802073G>T” (in Abstract , and page 10, paragraph 1 and page 11, paragraph 5). According to the contribution, the affiliation order was corrected: “Department of Clinical Pharmacology, Xiangya Hospital, Central South University” was corrected into the first affiliation.

These changes have no material impact on the conclusions of our paper. The manuscript will be updated and the original will remain online on the article webpage. We apologize for any inconvenience caused to our readers.
